# Phase 2 Randomized, Placebo-Controlled Clinical Trial of Recombinant Human Growth Hormone (rhGH) During Rehabilitation From Traumatic Brain Injury

**DOI:** 10.3389/fendo.2018.00520

**Published:** 2018-09-10

**Authors:** Rosemary Dubiel, Librada Callender, Cynthia Dunklin, Caryn Harper, Monica Bennett, Lisa Kreber, Richard Auchus, Ramon Diaz-Arrastia

**Affiliations:** ^1^Department of Physical Medicine and Rehabilitation, Baylor Institute for Rehabilitation, Dallas, TX, United States; ^2^Department of Neurology and Neurotherapeutics, University of Texas Southwestern Medical Center, Dallas, TX, United States; ^3^Center for Neuro Skills, Bakersfield, CA, United States; ^4^Department of Internal Medicine, University of Michigan, Ann Arbor, MI, United States; ^5^Department of Neurology, University of Pennsylvania, Philadelphia, PA, United States

**Keywords:** insulin-like growth factor I, glasgow outcome scale, disability rating scale, functional independence measure, short form 36

## Abstract

Traumatic brain injury (TBI) is a major cause of death and disability, but there are currently no therapies with proven efficacy for optimizing regeneration of repair during rehabilitation. Using standard stimulation tests, as many as 40–50% of survivors of severe TBI have deficiency of one or more pituitary hormones. Of these, the somatotropic axis is the most commonly affected, with Growth Hormone (GH) deficiency affecting ~20% of persons with severe TBI. Treatment with recombinant human Growth Hormone (rhGH) is generally effective in reversing the effects of acquired GH deficiency, but there is no evidence documenting functional or neurocognitive improvement after GH replacement in TBI patients. As a consequence, screening for GH deficiency and GH replacement when deficiency is found is not routinely performed as part of the rehabilitation of TBI survivors. Given that most of the recovery after TBI occurs within the first 6–12 months after injury and IGF-1 and GH are part of a coordinated restorative neurotrophic system, we hypothesized that patients will optimally benefit from GH therapy during the window of maximal neuroregenerative activity. We performed a Phase IIa, randomized, double-blind, placebo-controlled feasibility trial of recombinant human Growth Hormone (rhGH), starting at discharge from an inpatient rehabilitation unit, with follow up at 6 and 12 months. Our primary hypothesis was that treatment with rhGH in the subacute period would result in improved functional outcomes 6 months after injury. Our secondary hypothesis proposed that treatment with rhGH would increase IGF-1 levels and be well tolerated. Sixty-three subjects were randomized, and 40 completed the trial. At baseline, there was no correlation between IGF-1 levels and peak GH levels after L-arginine stimulation. IGF-1 levels increased after rhGH treatment, but it took longer than 1 month for levels to be higher than for placebo-treated patients. rhGH therapy was well-tolerated. The rhGH group was no different from placebo in the Disability Rating Scale, Glasgow Outcome Scale-Extended, or neuropsychological function. However, a trend toward greater improvement from baseline in Functional Independence Measure (FIM) was noted in the rhGH treated group. Future studies should include longer treatment periods, faster titration of rhGH, and larger sample sizes.

## Introduction

Traumatic brain injury (TBI) is a major cause of death and disability, with an estimated cost of 45 billion dollars a year in the United States alone. Every year, ~2.5 million people in the United States sustain a TBI, of which 53,000 people die, and another 284,000 are hospitalized and survive the injury (1). Currently, more than 5.3 million Americans (or 3% of the population) live with disabilities resulting from TBI and virtually no injury is without consequence (1). The magnitude of this problem has led to numerous clinical trials aimed at improving survival and/or functional outcome, yet no effective therapies have been identified. In particular, no therapies aimed at optimizing regeneration and repair during the rehabilitation period have been demonstrated to be effective.

TBI has long been recognized as a chronic cause of neuroendocrine dysfunction. While overt deficiency of the somatotropic, gonadotropic, thyroid, or adrenal axis have been recognized for many decades, subtler deficiencies have come to light only more recently. Using standard stimulation tests, as many as 40–50% of survivors of severe TBI have deficiency of one or more pituitary hormones (2). Of these, the somatotropic axis is the most vulnerable, with estimates of Growth Hormone (GH) deficiency affecting ~20% of persons with severe TBI (3). In contrast, thyroid hormone and adrenocorticotrophic hormone deficiency are relatively uncommon, with rates averaging 4–6%, while the gonadotrophic axis shows intermediate rates of dysfunction, between 8 and 12% (4–6). The clinical consequences of GH deficiency include decrease in muscle strength, decrease in lean body mass, and increase in abdominal fat, and reduced basal metabolic rate. However, the most common symptoms are neuropsychiatric, particularly fatigue, asthenia, depression, sleep disturbances, and memory problems (2). In a recently published study, GH deficiency, or insufficiency was associated with neurobehavioral deficits, decreased concentration, depression, and reduced quality of life (7) which underscores why GH deficiency after TBI is underdiagnosed as the symptoms and signs are non-specific and are often mistaken for or masked by the underlying traumatic injury.

Treatment with recombinant human Growth Hormone (rhGH) is generally effective in reversing the effects of acquired GH deficiency (8). In randomized controlled trials of adult patients with acquired GH deficiency (resulting primarily from pituitary tumors), rhGH therapy resulted in improved cognitive function, well-being, and quality of life (8, 9), in addition to improvements in metabolic factors such as bone mineral density and lean body mass. While studies have demonstrated the presence of GH deficiency in patients surviving TBI, there has been a paucity of evidence documenting functional or neurocognitive improvement after GH replacement therapy in TBI patients (10–13). A consequence of such lack of evidence is that screening for GH deficiency (and GH replacement when deficiency is found) is not routinely performed as part of the rehabilitation of TBI survivors.

Given that most of the recovery after TBI occurs within the first 6–12 months after injury and IGF-1 and GH are part of a coordinated restorative neurotrophic system, it is likely that they exert their effects in synergy with a host of other trophic and restorative factors that are maximally functioning within the first few months after injury. While treatment >1 year after injury in patients who remain GH deficient holds promise, we hypothesized that many patients will optimally benefit from GH therapy (and resulting elevations in IGF-1 levels) during the window of maximal neuroregenerative activity.

Treating only patients who have GH deficiency defined using stimulation tests (either glucagon or GHRH + L-arginine, which were developed for non-trauma populations), as was done in a past clinical trial, may be too conservative (14), as it may deny therapy to many patients with hypothalamic dysfunction and serum IGF-1 concentrations suboptimal to maximize recovery. It remains unclear what the optimal IGF-1 level is in patients recovering from TBI, and patients with serum IGF-1 levels in the lower end of the normal distribution may benefit from augmenting IGF-1 production with GH treatment, to maintain concentrations of this neurotrophic factor near the upper third of the normal range during the time window of maximal repair and regeneration.

This study is a Phase IIa, randomized, double-blind, placebo-controlled feasibility trial of rhGH, starting at discharge from an inpatient rehabilitation unit or while in a transitional rehabilitation setting, with follow up at 6 and 12 months. Our primary hypothesis was that treatment with recombinant human Growth Hormone (rhGH) in the subacute period after TBI would result in improved functional outcomes 6 months after injury as compared to placebo. Our secondary hypothesis proposed that treatment with rhGH would result in increased IGF-1 levels, that rhGH therapy would be well tolerated, and would not result in an increased rate of diabetes mellitus, arthralgias, or peripheral edema.

## Methods

Participants who suffered acute TBI were recruited into the study from an acute inpatient rehabilitation or patients receiving care at a transitional post-acute rehabilitation setting. Our goal was to enroll participants who suffered a sufficiently severe injury to require inpatient rehabilitation or transitional rehab care and were at a timeframe with the most potential for neuroregeneration. Table 1 details eligibility criteria used for screening. This study was approved by the Institutional Review Boards at Baylor University Medical Center and the University of Texas Southwestern Medical Center, both in Dallas, Texas. Written consent was obtained from all participants or their legally authorized representatives, when the participant did not have capacity to provide informed consent. In these latter cases assent was obtained from the participant. The trial was registered with ClinicalTrials.gov (NCT 00766038) prior to the first enrollment.

**Table 1:** Inclusion and exclusion criteria.

**Inclusion criteria**	**Exclusion criteria**
1. Non-penetrating TBI	1. History of pre-existing neurologic disease (such as brain tumors, meningitis, cerebral palsy, encephalitis, brain abscesses, vascular malformations, cerebrovascular disease, Alzheimer's disease, multiple sclerosis, or HIV-encephalitis)
2. Age 16–65 years	2. History of premorbid disabling condition that interfere with outcome assessments
3. Randomization within 2–24 weeks of injury.	3. Contraindication to rhGH therapy (hypersensitivity to rhGH or any of the components of the supplied product, including metacresol, glycerin, or benzyl alcohol)
4. Rancho Los Amigos Rating IV or better at the time of randomization. Should not be at Rancho IV level for more than 18 weeks before randomization	4. Penetrating traumatic brain injury
5. Availability of caregiver to oversee administration of medications	5. Discharged from rehabilitation on insulin therapy and/or hypoglycemic agents
6. Reasonable expectation for completion of outcome measures	6. Obesity (BMI > 35)
7. Residence inside the United States	7. Active infection
	8. Hypothyroidism or Adrenal Insufficiency
	9. Previous diagnosis of renal or hepatic failure
	10. Active malignant disease
	11. Acute critical illness, heart failure, or acute respiratory failure
	12. Membership in a vulnerable population (prisoner)
	13. Pregnancy. Women of childbearing age will be given a pregnancy test during screening to exclude pregnancy
	14. Lactating females

### Subjects

**Figure 1:**
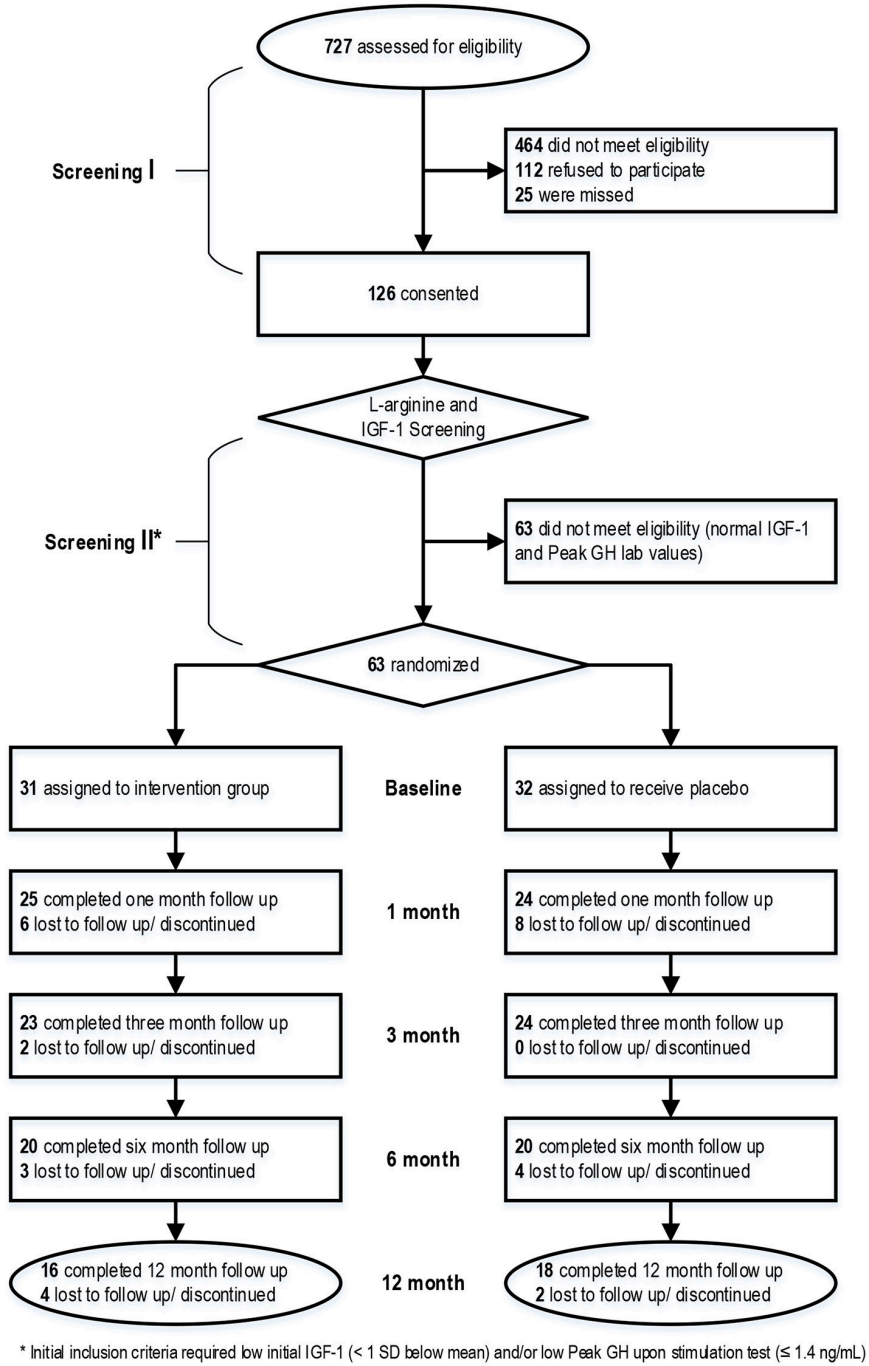
CONSORT diagram showing the flow of participants from screening through study completion.

Figure 1 shows a CONSORT diagram which shows the flow of patient activity from screening through completion of the project. Informed consent was obtained from all participants before any study activities were conducted.

A total of 63 subjects out of 126 subjects who consented were eligible for randomization into the study with 31 randomized to treatment with rhGH and 32 randomized to placebo. IGF-1 levels were measured on all patients with some patients undergoing dynamic GH testing. Initial inclusion criteria required low initial IGF-1 (<1 standard deviation below mean) and/or a low Peak GH upon stimulation (</= 1.4 ng/mL). Age and gender-specific reference ranges (mean and SDs) were taken from published tables (15). Due to a national unavailability of intravenous L-arginine which developed during the study, dynamic stimulation testing was not available for all participants. Eligible patients were randomized in a double-blind fashion to (Group 1) rhGH subcutaneously or (Group 2) placebo. The vehicle for placebo treatment was normal saline, packaged in multi-dose injectors identical to the rhGH given to the treatment group. The GH treatment arm received a starting dose of 400 μg/day, with increases (or decreases) in dose by 100–200 μg/day based on IGF-1 labs results obtained at 1 and 3 months, until target IGF-1 (in the upper quintile of the range for age and body weight) was reached up to maximum dose of 1,000 μg/day. Doses for participants receiving placebo were also adjusted monthly to maintain the blinding. Treatment continued for a period of 6 months.

Treatment was overseen by a board certified endocrinologist (RA) according to practice guidelines released by the Endocrine Society “Clinical Guidelines for Evaluation and Treatment of Adult Growth Hormone Deficiency” (16, 17). In this study, to accommodate both GH-deficient and GH-sufficient strata, the treatment goal was serum IGF-1 as close as possible to the upper limit for the age-adjusted reference range without exceeding the normal range. The dose of GH (or placebo) was adjusted by the study physician depending on the occurrence and severity of adverse effects.

### Safety assessments

Safety was assessed by clinical evaluations at 1, 3, and 6 and 12 months after initiating therapy. At these evaluations participants and their caregivers were asked about possible adverse effects of GH treatment, including fluid retention, paresthesias, joint stiffness, peripheral edema, arthralgias, and myalgias. Additionally, blood was obtained for assessment of glucose, free T4, lipid profile, and insulin level.

Study visits and procedures at each visit are depicted in Table 2.

**Table 2:** Procedures at each visit.

**Procedure**	**Baseline**	**1 month**	**3 months**	**6 months**	**12 months**
Informed consent	X				
Education on injections	X	X	X		
Glascow Outcome Scale Extended (GOS-E)			X	X	X
Disability Rating Scale (DRS)	X		X	X	X
Functional Independence Measure (FIM)	X		X	X	X
Neuropsych. battery				X	X
L-Arginine stimulation test	X				X
Glucose, free T4, lipid profile, and insulin	X	X	X	X	X
Serum Insulin-like Growth Factor-1 (IGF-1) levels	X	X	X	X	X
Medication compliance		X	X	X	

### Functional outcomes and neuropsychological testing

An abridged neuropsychological battery was conducted at baseline, with additional tests at 6 and 12 months (Table 2). These measures are designed to capture a broad perspective of functional and cognitive parameters that are important following TBI, and utilizes scales that have a long history in TBI research. Further, there is convincing evidence that they can be reliably administered 6–12 month after injury. Functional Independence Measure (FIM) was tracked at baseline, 3, 6, and 12 months as a measure of the severity of functional impairment, during the course of study enrollment. FIM consists of 18 items, with each item rated on a 7-point scale, ranging from 1 (total assistance) to 7 (complete independence). Global function was measured by the Disability Rating Scale (DRS) and Glasgow Outcome Scale—Extended (GOSE). Cognitive executive function was measured with the Galveston Orientation and Amnesia Test (GOAT), Digit Span and Processing Speed Index from the WAIS-III, the DKEFS Stroop test, Trail Making Test—Part A and B, California Verbal Learning Test-2 (CVLT-2), and Controlled Word Association (COWA). Emotional behavior function was measured with the Brief Symptom Inventory (BSI), Fatigue Severity Scale (FSS), and the Rivermead Post Concussion Symptom Questionnaire. Finally, quality of life was assessed using the Satisfaction with Life Scale (SWLS) and the Short-Form 36 (SF-36).

### Biochemical tests

IGF-1 levels were measured by Quest Laboratories using a liquid chromatrography/mass spectrometry assay. This laboratory also carried out the safety lab assessments (glucose, insulin, lipid profile). See legend at bottom of Table **7** for normative lab values.

### Statistical analysis

In this Phase II study, for the primary hypothesis, the design of our study was that of a non-futility study, powered to not reject a potentially useful therapy, rather than prove efficacy. All participants in the GH treatment arm did not achieve goal serum IGF-1 values in the first month, data was analyzed in an intention-to-treat manner.

Final analysis included 40 subjects who completed 6 month follow up measures. Baseline characteristics for the treatment and placebo groups were compared using *t-*tests or Mann–Whitney-*U*-test for numerical variables and chi-square or Fisher's exact tests for categorical variables. To compare IGF-1 levels between groups at each visit, Mann–Whitney *U*-tests were used. Other functional and neuropsychological outcomes listed in Table 2 were compared at both the 6-month visit and the 12 month visit. *T*-tests, Mann–Whitney *U*-tests or chi-square tests were performed, as appropriate, to determine if there were significant differences in each outcome. Comparison of adverse events between treatment groups were performed using Fisher's exact tests due to low counts. To determine if participants who withdrew or were lost to follow-up were different than those who completed the 6 month visit, baseline characteristics were compared using the same tests mentioned above. All analyses were performed using SAS 9.4 with a 5% significance level of 5%.

## Results

Sixty-three subjects were randomized into the study, 31 in the rhGH intervention group and 32 in the placebo group. Of those randomized, 40 subjects (20 in each group) completed treatment through the 6 month follow up visit. Table 3 shows the baseline characteristics of those participants who completed 6 months of treatment, stratified by treatment and placebo groups. On average, subjects were 31.1 ± 14.3 years old, male (85%), and White (92.5%). There were no significant differences between the intervention and placebo group except in cause of injury and FIM admission scores.

**Table 3:** Baseline characteristics by treatment group and overall.

	**All with 6 month f/u** **(*n* = 40)**	**rhGH** **(*n* = 20)**	**Placebo** **(*n* = 20)**	***p*-value**
**Age** (years), mean (STD)	31.1 (14.3)	32.2 (15.2)	30.1 (13.7)	0.656
**Gender**, Male, *n* (%)	34 (85)	17 (85)	17 (85)	1.00
**Ethnicity**, Hispanic, *n* (%)	6 (15)	3 (15)	3 (15)	1.00
**Race**, White, *n* (%)	37 (92.5)	18 (90)	19 (95)	0.548
**Injury Mechanism**				**0.037**
Motor vehicle, *n* (%)	18 (45)	5 (25)	13 (65)	
Other vehicular, *n* (%)	15 (37.5)	9 (45)	6 (30)	
Fall, *n* (%)	3 (7.5)	3 (15)	0 (0)	
Other, *n* (%)	4 (10)	3 (15)	1 (5)	
**Days from injury to randomization**, mean (STD)	64.1 (35.9)	65.7 (30.4)	62.5 (41.4)	0.782
Severity as measured by Glascow Coma Score (GCS) and Post-traumatic Amnesia (PTA)^*^				0.186
Mild, *n* (%)	4 (10)	3 (15)	1 (5)	
Moderate, *n* (%)	3 (7.5)	0 (0)	3 (15)	
Severe, *n* (%)	32 (80)	17 (85)	15 (75)	
Unknown/Missing, *n* (%)	1 (2.5)	0 (0)	1 (5)	
**Disability Rating Scale (DRS)**, mean (STD)	8 (4.1)	7.6 (3)	8.4 (5.1)	0.546
**FUNCTIONAL INDEPENDENCE MEASURE (FIM) ADMIT**				
Motor, mean (STD)	38.3 (18.4)	31.7 (17.4)	44.3 (17.6)	**0.033**
Cognitive, mean (STD)	9.3 (6.0)	7.3 (4.8)	11.1 (6.6)	0.053
Total, mean (STD)	47.7 (22.2)	39.1 (20.3)	55.4 (21.5)	**0.022**
**FIM DISCHARGE**				
Motor, mean (STD)	79.0 (22.0)	80.0 (23.0)	78.0 (21.5)	0.784
Cognitive, mean (STD)	23.9 (8.1)	23.2 (7.1)	24.6 (9.1)	0.590
Total, mean (STD)	102.9 (28.4)	103.2 (28.0)	102.6 (29.5)	0.952
**FIM CHANGE**				
Motor, mean (STD)	39.0 (17.1)	44.5 (17.9)	33.7 (14.9)	**0.043**
Cognitive, mean (STD)	14.5 (7.3)	15.5 (6.7)	13.5 (7.9)	0.408
Total, mean (STD)	53.5 (22.2)	60.4 (21.0)	47.2 (21.8)	0.066
**IGF-1 below age and gender specific mean**, *n* (%)	21 (54)	12 (60)	9 (47.7)	0.525
**IGF-1**<**1 STD below mean**, n (%)	6 (15.4)	3 (15.8)	3 (15)	0.946
**Peak L-Arginine** (*n* = 24), median [IQR]	1.3 [0.5, 5.8]	2.2 [0.6, 5.9]	1.2 [0.5, 5.7]	0.395
**CVLT Score**, mean (STD)	29.4 (17.0)	25.4 (18.7)	33.4 (14.9)	0.093
**CVLT t-score**, mean (STD)	30.5 (17.8)	27.2 (18.6)	34 (16.9)	0.159
**Trails A Score**, mean (STD)	54.7 (23.7)	57.8 (25.9)	51.9 (22.1)	0.462
**Trails A t-score**, mean (STD)	26.5 (14.6)	26 (16.3)	27.1 (13.4)	0.908
**Trails B Score**, mean (STD)	156.4 (97.9)	142 (106.8)	167.8 (92.7)	0.603
**Trails B t-score**, mean (STD)	29.7 (18.3)	28.7 (19.4)	30.5 (18.1)	0.908

* *Severity defined according to Mayo classification system: Malec et al. (18). Bold values highlight those that meet the pre-specified nominal threshold for statistical significance (p < 0.05)*.

**Figure 2:**
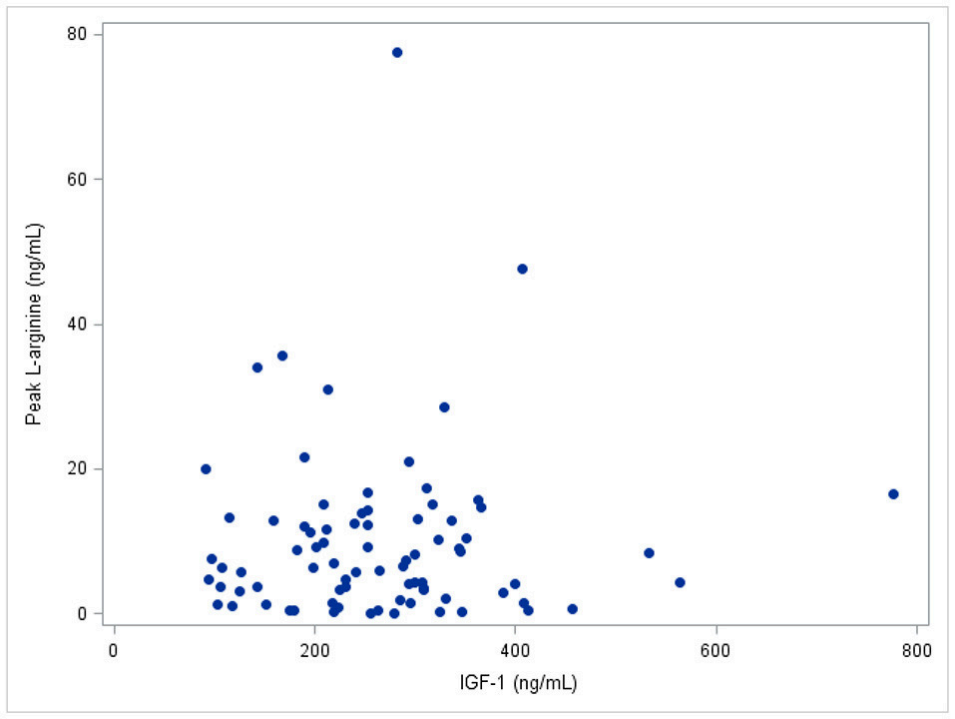
IGF-1 levels vs. peak L-arginine level. The Pearson correlation between IGF-1 and peak L-arginine is 0.03, *p* > 0.2.

Only 24 participants were underwent dynamic GH testing (Table 3), due to the unforeseen unavailability of L-arginine mid-way through the study. Figure 2 shows a scatterplot that depicts there was no correlation between IGF-1 levels and peak GH levels after L-arginine stimulation.

Table 4 shows a crude analysis of IGF-1 levels between the treatment and placebo groups. There was a significant difference between the two groups at 3 and 6 months (*p* < 0.05), but not after 1 month of treatment. The lack of difference between the 2 groups at 1 month was expected, as the initial dose of rhGH chosen was relative low and time was required to adjust the dose of rhGH properly to therapeutic levels based on measured drug levels. As expected, there were no longer differences in IGF-1 levels at 12 months since treatment with either drug or placebo ended 6 months.

**Table 4:** IGF-1, crude analysis.

**Visit**	**rhGH, median [IQR]**	**Placebo, median [IQR]**	***p-*value**
Baseline	218.5 ng/mL [147.0, 263.5]	194.0 ng/mL [151.0, 279.0]	0.911
1 Month	272.5 ng/mL [212.5, 354.0]	246.0 ng/mL [197.0, 312.0]	0.337
3 Month	282.0 ng/mL [183.0, 377.0]	213.0 ng/mL [158.0, 259.0]	0.035
6 Month	245.5 ng/mL [203.5, 321.5]	173.0 ng/mL [145.0, 237.0]	0.005
12 Month	185.5 ng/mL [143.0, 252.0]	172.5 ng/mL [148.0, 222.0]	0.836

*2-sided p-value from PROC NPAR1WAY Wilcoxon Rank Sum Test; Baseline through 6 Month contain only participants with 6 month follow-up (n = 40; 20 each group); 12 month contains only participants with 12 month follow-up (n = 16 for rhGH, n = 18 for placebo)*.

Tables 5, 6 show the primary outcomes, including neuropsychological testing and functional outcomes, stratified by the treatment and placebo groups. There were no significant differences seen between the two groups for any of the neuropsychological measures or DRS. While neuropsychological testing was conducted at 3 months, the results are not shown in table form as they are consistent with the results at 6 and 12 months, showing no statistically significant differences. However, there was variation in FIM scores, with the rhGH treatment group showing significantly greater change for FIM motor, FIM cognitive, and FIM total from baseline to 6 month follow up (*p* = 0.02 for all strata). At 12 month follow up FIM motor change and FIM total change were significantly greater in the rhGH treatment group compared to the placebo group (*p* = 0.02 and *p* = 0.01, respectively). There was no correction for multiple comparisons.

**Table 5:** Selected outcomes at 6 months.

	**Overall,** ***n* = 40 median [IQR]**	**rhGH** ***n* = 20 median [IQR]**	**Placebo** ***n* = 20 median [IQR]**	***p*-value**
Disability Rating Scale (DRS)	1.5 [0.0, 4.0]	2.0 [0.0, 4.5]	1.0 [0.0, 3.5]	0.59
Galveston Orientation Assessment Test (GOAT)	99.5 [90.0, 100]	98.5 [92.5, 100]	99.5 [89.0, 100]	1.0
Digit span age standardized score	9.0 [6.0, 13.0]	9.0 [6.5, 12.0]	9.0 [6.0, 13.0]	0.84
Processing speed index age standardized score	84.0 [73.0, 99.0]	82.5 [73.0, 96.0]	88.0 [79.0, 99.0]	0.80
Digit symbol age standardized score	7.0 [5.0, 9.0]	6.0 [4.0, 9.0]	7.0 [6.0, 9.0]	0.31
Symbol search age standardized score	8.0 [6.0, 11.0]	7.0 [5.0, 11.0]	8.0 [7.0, 11.0]	0.44
California Verbal Learning Test (CVLT) T score	41.5 [32.0, 55.0]	38.0 [32.0, 59.0]	42.0 [32.0, 55.0]	0.81
Trails A T score	42.0 [34.0, 51.0]	39.5 [19.5, 53.5]	43.0 [40.0, 50.0]	0.34
Trails B T score	47.0 [33.0, 56.0]	47.0 [29.5, 53.5]	47.0 [35.0, 56.0]	0.46
Controlled Oral Word Association Test (COWA) T score	34.0 [26.0, 44.0]	31.0 [24.5, 45.5]	36.0 [33.0. 42.0]	0.26
Brief Symptom Inventory (BSI)—Average T score	51.2 [43.9, 59.2]	51.6 [46.9, 59.6]	48.8 [43.9, 58.6]	0.89
Functional Independence Measure (FIM) Motor	110.0 (17.9)	112.2 (12.9)	107.8 (22.0)	0.82
FIM cognitive	31.5 (5.3)	31.9 (3.7)	31.2 (6.7)	0.89
FIM total	141.5 (21.9)	144.1 (14.3)	139.0 (27.6)	0.99
FIM motor change from baseline	71.3 (22.3)	80.1 (18.9)	63.5 (22.6)	**0.02**
FIM cognitive change from baseline	22.3 (7.1)	24.7 (6.0)	20.1 (7.4)	**0.02**
FIM total change from baseline	93.6 (26.6)	104.8 (20.8)	83.6 (27.7)	**0.02**
Patient Health Questionnaire (PHQ-9)	9.8 (19.6)	6.5 (7.5)	13.2 (26.7)	0.85
Fatigue severity scale	26.5 (14.9)	23.6 (13.0)	29.5 (16.6)	0.27
Rivermead post-concussion symptoms questionnaire	14.4 (13.9)	15.2 (15.0)	13.6 (13.0)	0.78
Satisfaction with life scale	21.2 (9.5)	22.2 (10.2)	20.3 (9.0)	0.56
Short Form health survey (SF 36)—physical health	266.6 (57.8)	274.6 (50.1)	258.3.7 (65.4)	0.74
Short Form health survey (SF 36)—mental health	246.2 (55.4)	260.3 (55.4)	231.3 (52.8)	0.10
**6 month Glascow Outcome Score-Extended (GOSE) score**				0.72
3—Severe disability	6	3	3	
4	6	5	1	
5—Moderate disability	4	2	2	
6	5	1	4	
7—Good recovery	11	4	7	
8	8	5	3	

*2-sided p-value (α = 0.05) from PROC NPAR1WAY Wilcoxon Rank sum test. Bold values highlight those that meet the pre-specified nominal threshold for statistical significance (p < 0.05)*.

**Table 6:** Selected outcomes at 12 months.

	**Overall, *n* = 34 median [IQR]/mean (sd)**	**rhGH *n* = 16 median [IQR]/mean (sd)**	**Placebo *n* = 18 median [IQR]/mean (sd)**	***p*-value**
Disability Rating Scale (DRS)	0.0 [0.0, 4.0]	0.5 [0.0, 3.0]	0.0 [0.0, 4.0]	0.73
Galveston Orientation Assessment Test (GOAT)	100 [87.0, 100]	99.5 [91.0, 100]	100 [87.0, 100]	0.93
Digit span age standardized score	11.0 [8.0, 13.0]	10.5 [8.0, 14.5]	11.0 [9.0, 13]	0.93
Processing speed index age standardized score	88.0 [79.0, 99.0]	86.0 [76.0, 111]	89.5 [81.0, 99.0]	0.90
Digit symbol age standardized score	8.0 [6.0, 9.0]	8.0 [6.0, 12.0]	8.0 [6.0, 9.0]	0.68
Symbol search age standardized score	9.0 [6.0, 11.0]	9.0 [6.0, 12.0]	9.0 [7.0, 11.0]	0.97
California Verbal Learning Test (CVLT) T score	43.5 [32.0, 59.0]	41.0 [28.5, 62.0]	47.0 [41.0, 59.0]	0.37
Trails A T score	49.0 [44.0, 55.0]	47.0 [26.0, 56.0]	51.0 [44.0, 55.0]	0.56
Trails B T score	52.0 [46.0, 62.0]	50.0 [35.0, 62.0]	54.0 [48.0, 62.0]	0.38
Controlled Oral Word Association Test (COWA) T score	38.0 [30.0, 47.0]	33.0 [22.0, 47.0]	38.0 [34.0. 49.0]	0.30
Brief Symptom Inventory (BSI)—Average T score	50.3 [45.5, 60.9]	56.6 [44.8, 63.0]	49.6 [47.9, 56.6]	0.82
Functional Independence Measure (FIM) Motor	113.5 (14.9)	115.9 (8.0)	111.4 (19.1)	0.56
FIM cognitive	31.7 (4.3)	32.2 (3.3)	31.3 (5.2)	0.87
FIM total	142.5 (17.5)	148.1 (9.5)	142.7 (22.4)	0.85
FIM motor change from baseline admit	74.8 (21.5)	83.7 (18.6)	66.8 (21.2)	**0.02**
FIM cognitive change from baseline admit	22.7 (6.1)	24.9 (5.6)	20.8 (6.0)	0.05
FIM total change from baseline admit	97.5 (25.1)	108.6 (20.9)	87.6 (24.9)	**0.01**
Patient Health Questionnaire (PHQ-9)	4.7 (6.0)	6.2 (7.4)	3.4 (4.2)	0.54
Fatigue severity scale	25.4 (14.0)	24.3 (13.8)	26.5 (14.6)	0.54
Rivermead post-concussion symptoms questionnaire	11.1 (10.6)	11.1 (9.9)	11.1 (11.5)	0.86
Satisfaction with life scale	22.7 (8.1)	23.4 (8.5)	22.1 (8.0)	0.58
Short Form health survey (SF 36)—Physical health	277.7 (31.9)	287.8 (33.8)	268.1 (27.6)	0.08
Short Form health survey (SF 36)—Mental health	245.7 (48.0)	244.9 (59.2)	246.4 (36.3)	0.54
**12 month Glascow Outcome Score-Extended (GOSE) score**				0.59
3—Severe disability	4	1	3	
4	4	3	1	
5—Moderate disability	1	0	1	
6	8	4	4	
7—Good recovery	5	1	4	
8	12	7	5	

*2-sided p-value (α = 0.05) from PROC NPAR1WAY Wilcoxon Rank sum test. Bold values highlight those that meet the pre-specified nominal threshold for statistical significance (p < 0.05)*.

There was no difference among safety labs between rhGH and placebo groups as outlined in Table 7. Finally, adverse events between the rhGH and placebo groups for all 63 participants are described in Table 8. Two participants in the rhGH group and 3 participants in the placebo group discontinued the study drug and participation in the project due to adverse events. There was no statistically significant difference in the reporting of undesirable side effects in the treatment group compared to the placebo group.

**Table 7:** Comparison of safety lab values.

	**rhGH,** **mean (sd)**	**Placebo,** **mean (sd)**	***p*-value**
**HDL CHOLESTEROL**			
Baseline	39.9 (11.7)	37.7 (10.2)	0.84
1 Month	49.3 (13)	43.3 (10.5)	0.13
3 Month	48.3 (13.3)	42.2 (9.5)	0.20
6 Month	47.3 (10.6)	44.4 (8.6)	0.45
12 Month	48.9 (14.8)	41.3 (13.9)	0.23
**LDL CHOLESTEROL**			
Baseline	108.1 (33.3)	109.7 (29.6)	0.54
1 Month	109.9 (26.8)	112.7 (33.7)	0.66
3 Month	110.2 (35.1)	100.6 (31)	0.35
6 Month	108.9 (34.9)	102.4 (40.2)	0.46
12 Month	108.9 (32.2)	109.9 (35)	0.93
**TOTAL CHOLESTEROL**			
Baseline	172.9 (41)	176.6 (40.2)	0.76
1 Month	183.8 (32.8)	184.5 (39.1)	0.88
3 Month	188.2 (43.9)	177.9 (36.9)	0.35
6 Month	182.5 (40.6)	187.4 (42.5)	0.97
12 Month	186.6 (42.5)	184.2 (32.5)	0.74
**TRIGLYCERIDES**			
Baseline	127.5 (64.1)	147.3 (67.3)	0.23
1 Month	123 (48.4)	141.9 (56)	0.23
3 Month	150.7 (100.9)	175.6 (91.9)	0.29
6 Month	130.8 (72.2)	192.5 (139.8)	0.17
12 Month	143.8 (82.5)	164.9 (80.2)	0.38
**GLUCOSE**			
Baseline	84.4 (9.2)	86 (8.3)	0.42
1 Month	69.7 (25.3)	80 (16.2)	0.27
3 Month	83.6 (22.1)	90.2 (20.5)	0.41
6 Month	88.7 (15.8)	84.5 (10.3)	0.25
12 Month	88.3 (10.8)	89.1 (12.1)	0.90
**t4, FREE**			
Baseline	1.6 (2.1)	1.1 (0.3)	0.89
1 Month	1.4 (1.6)	1.1 (0.3)	0.99
3 Month	1.7 (2.2)	1.1 (0.2)	0.33
6 Month	1.9 (2.3)	1.2 (0.2)	0.90
12 Month	1.2 (0.2)	1.2 (0.2)	0.86
**INSULIN**			
Baseline	3.2 (3.1)	7.1 (9.5)	0.21
1 Month	5.9 (9.9)	5.4 (5.9)	0.34
3 Month	11.9 (20)	11.1 (14.7)	0.44
6 Month	9.8 (15.1)	8.4 (8.9)	0.38
12 Month	7.3 (12.9)	16.9 (16.9)	0.04

All laboratory diagnostics were performed via Quest Diagnostics. Normal lab value references: Cholesterol, total 125–200 mg/dL; HDL Cholesterol > or + 40 mg/dL; Triglycerides <150 mg/dL; LDL Cholesterol <130 mg/dL; Glucose 65–99 mg/dL; T4, free 0.8–1.8 ng/dL; Insulin <17 uIU/mL; Growth hormone: Using the GH stimulation test, the following results would rule out GH deficiency: Adults (> or + 20 years) – Arginine/GHRH > or = 5.1 ng/mL, Children (<20 years) – Arginine/GHRH > or = 10 ng/mL; IGF 1 50–303 ng/mL.

*rhGH n = 20 at baseline, 3 and 6 months, n = 16 at 12 months; placebo n = 20 at baseline, 3 and 6 months, n = 18 at 12 months*.

**Table 8:** Comparison of adverse events.

	**All (*n* = 63)**	**rhGH** **(*n* = 31)**	**Placebo** **(*n* = 32)**	***p*-value**
None reported	36 (57.1)	19 (61.3)	17 (53.1)	
Arthralgia	8 (12.7)	2 (6.5)	6 (18.8)	0.256
Myalgia	7 (11.1)	1 (3.2)	6 (18.8)	0.104
Headache	4 (6.3)	1 (3.2)	3 (9.4)	0.613
Elective surgery	10 (15.9)	6 (19.4)	4 (12.5)	0.509
Urinary issue	3 (4.8)	0 (0)	3 (9.4)	0.238
Back pain	1 (1.6)	1 (3.2)	0 (0)	0.492
Broken ribs and scapula	1 (1.6)	0 (0)	1 (3.1)	1.0
Bruising at injection site	1 (1.6)	1 (3.2)	0 (0)	0.492
Drowsiness	2 (3.2)	1 (3.2)	1 (3.1)	1.0
Dizziness	1 (1.6)	1 (3.2)	0 (0)	0.492
Dry mouth	4 (6.3)	1 (3.2)	3 (9.4)	0.613
Eye puffiness	1 (1.6)	0 (0)	1 (3.1)	1.0
Hair growth	1 (1.6)	1 (3.2)	0 (0)	0.492
Hair loss	1 (1.6)	0 (0)	1 (3.1)	1.0
Weight gain	1 (1.6)	0 (0)	1 (3.1)	1.0
Irritability	1 (1.6)	0 (0)	1 (3.1)	1.0
Numbness in extremities	1 (1.6)	0 (0)	1 (3.1)	1.0
Peripheral edema	1 (1.6)	0 (0)	1 (3.1)	1.0
Seizure	2 (3.2)	2 (6.5)	0 (0)	0.613
**Greatest Action Taken**				0.317
Not Applicable (N/A)	36 (57.1)	19 (61.3)	17 (53.1)	
None required	15 (23.8)	9 (29)	6 (18.8)	
Treatment given	7 (11.1)	1 (3.2)	6 (18.8)	
Study drug temporarily stopped	2 (3.2)	0 (0)	2 (6.3)	
Study drug discontinued	3 (4.8)	2 (6.5)	1 (3.1)	

## Discussion

There is extensive data from experimental models that GH and IGF-1 are part of the neuro-restorative response after TBI and other acquired brain injuries, such as ischemic stroke, hypoxia-ischemia, and excitotoxins. Treatment with rhGH (19–24) or IGF-1 (19, 25–27) results in improved neurological and cognitive outcome in multiple experimental models. This is particularly the case when therapy is administered early after injury (21, 23). In humans, higher levels of IGF-1 are associated with greater white matter recovery after TBI (16), and improved functional outcome after ischemic stroke (28, 29). These well-established observations formed the scientific rationale for this study.

While GH deficiency is a well-recognized consequence of TBI, there is little Class I evidence that rhGH replacement produces clinical benefits. A consequence of such lack of evidence is that screening for GH deficiency (and GH replacement when deficiency is found) is not routinely performed as part of the rehabilitation of TBI survivors. Several small clinical trials provide useful information on the issue of whether replacement therapy with rhGH is clinically beneficial in chronic post-TBI patients (>1 year after injury). High et al. (11) randomized 23 subjects to either GH replacement or placebo. Despite the small sample size, this study clearly showed that GH replacement therapy is effective in increasing serum IGF-1 levels in GH deficient and insufficient subjects, defined as a GH peak under 3 or 8 ng/ml (respectively) upon glucagon stimulation. There was also an indication that GH replacement was associated with improvement in several neuropsychological measures. Moreau et al. (12) conducted an open-label study on 23 TBI patients with GH deficiency (average 7.8 years after injury), compared to 27 controls (not treated with rhGH because they had normal or near-normal somatotropic function (*n* = 24) or who had contra-indications or refused treatment with rhGH. There were benefits in cognitive function, particularly in attention, memory, and visuospatial abilities. Reimunde et al. (13) treated 11 patients (average 3.7 years after injury) with GH deficiency with rhGH, and compared them with 8 controls without GH deficiency, and found evidence of greater improvement in several cognitive tests in the rhGH treated group. Devesa et al. (10) reported treated 13 TBI patients (2.5 months to 11 years after injury) with rhGH; 5 patients had GH deficiency and 8 did not. In this open label study inconsistent cognitive improvements were noted.

For the primary hypothesis, the treatment group had significantly greater FIM motor change and FIM total change from baseline that the placebo group at 6 and 12 months. This must be interpreted with caution given that the treatment group had a significant lower FIM motor and total FIM score at baseline as compared to the placebo group. However, the treatment group did achieve a statistically significant improvement in the FIM cognitive change at 6 months despite the two groups having similar FIM cog scores at baseline. However, there was no significance seen in the FIM cognitive change at 12 months that was seen at 6 months testing. The most likely reason for FIM change significance is the imbalance of the functional measures at entry to the study and the possible statistical artifact with regression of the treatment group toward the mean. Additionally, there was no statistically significant difference in the FIM motor, FIM cognitive or FIM total scores between the two groups at either time period.

Our key secondary analyses did show increased IGF-1 levels in those treated with rhGH, however the first measure where IGF-1 levels statistically differed from the placebo group was not seen until month 3. The fact that this effect took at least 3 months to manifest was likely due to the design of the study in which subjects were started at lower rhGH doses which were likely to be well-tolerated. It is possible that more aggressive initial dosing, faster titration of dosing, or longer period of treatment may be necessary to see an observable impact on functional outcomes.

Patients treated with rhGH did achieve higher IGF-1 levels that those receiving placebo, with significant increases seen at 3 and 6 months with IGF-1 levels returning to pre-treatment levels by 12 months. At the 12-month timeframe, both groups had similar IGF-1 levels, indicating that IGF-1 does not stay elevated after cessation of treatment. Clinically this could hold promise since IGF-1 is a putative neuroregenerative factor after TBI. Recent preclinical and clinical data suggests that maintenance of high IGF-1 levels would be beneficial after TBI. IGF-1 is a neurotrophic factor (30), both in experimental models (27) as well as in human diseases such as Alzheimer's disease and vascular dementia (31). It appears that IGF-1 is part of a neurotrophic response to multiple types of injury to the CNS, including TBI (32). In rodents, hypoxic-ischemic injury is ameliorated by recombinant IGF-1 administration (25), regardless of whether the drug is administered 2 h after or 1 h before injury. In the cortical stab injury model, blockade of IGF-1 by an anti-IGF-1 antibody reduces neovascularization at the wound site (30). In the same model, administration of IGF-1 4 and 12 h post-injury resulted in improved motor activity and decrease in apoptotic cells in the peritraumatic area (26). In the fluid percussion model in rats, administration of IGF-1 starting 15 min postinjury and continuing for 14 days resulted improved neuromotor function and enhanced learning ability, which was evident 14 days after injury but not at 2 or 7 days (27).

There have been studies that found greater incidence of depression and decreased quality of life in GH-deficient subjects (7). While no significance was reached in clinical outcomes at 6 and 12 months, there was a trend in the rhGH treated group to have higher SF 36 Physical health verse the placebo group at 6 months and another trend was seen in the rhGH treated group to having higher scores on the SF 36 mental health at 12 months. Additionally, it is important to note that the majority of those in the study (both drug and placebo groups) achieved relatively good functional outcomes, with high moderate disability to good recovery noted on the Glasgow Outcome Scale-Extended (GOSE) by 12 months. It is possible that rhGH would have a more favorable effect in more severely injured subjects, destined for less favorable recovery.

We were able to show that treatment with rhGH was overall well-tolerated in subjects with no significant side effects compared to placebo in developing rates of undesirable side effects that can occur with rhGH replacement. In fact, comparison of adverse event data revealed that there were only 2 events in the treatment group requiring temporary cessation or discontinuation of the study drug compared to 3 such events in the placebo group.

There are limitations that need mentioning. Due to low power based on smaller than projected enrollment and a moderate number of dropouts, we were not able to show that IGF-1 is a prognostic biomarker (meaning that those that have a higher baseline IGF-1 have improved outcomes). Additionally, analysis of the data in only male subjects in order to address sex differences in regards to treatment effect was not able to be performed due to lack of sufficient power. Another limitation of our study was the lack of all patients having dynamic testing of GH. While there are studies that report IGF-1 levels to be highly predictive of GH deficiency (5, 33), GH levels are most routinely assessed following stimulation with arginine, glucagon, or an insulin tolerance test. Due to national shortage of L-arginine during the study, not all patients were assessed with dynamic testing methods.

## Conclusions

This phase IIa, randomized, double-blind, placebo-controlled trial showed that it is feasible to achieve IGF-1 levels in the upper quartile in patients following severe TBI in the subacute period, and that treatment with rhGH is well-tolerated. While the rhGH treatment group did achieve statistically greater FIM total and FIM motor change than the placebo group, it is impossible to ascribe such change to rhGH treatment, since the relatively low sample size made it impossible to adjust for multiple comparisons and possible confounders. Future studies should look at longer treatment periods, consider faster titration of rhGH during the treatment period as well as seek further evidence in supporting rhGH treatment effect on clinical outcomes.

## Author contributions

RD, LC,CD, CH, LK, MB, and RD-A contributed to the design of the study, participated in patient enrollment and followup, and was involved in data analysis and preparation or manuscript for publication. RA participated in study design and adjusted medication doses in a blinded fashion.

### Conflict of interest statement

The authors declare that the research was conducted in the absence of any commercial or financial relationships that could be construed as a potential conflict of interest.
